# Crystal structure of 2-(11-oxo-10*H*,11*H*-indeno­[1,2-*b*]chromen-10-yl)-2,3-di­hydro-1*H*-indene-1,3-dione

**DOI:** 10.1107/S2056989015007495

**Published:** 2015-04-22

**Authors:** Joel T. Mague, Shaaban K. Mohamed, Mehmet Akkurt, Antanr A. Abdelhamid, Mustafa R. Albayati

**Affiliations:** a Department of Chemistry, Tulane University, New Orleans, LA 70118, USA; b Chemistry and Environmental Division, Manchester Metropolitan University, Manchester M1 5GD, England; c Chemistry Department, Faculty of Science, Minia University, 61519 El-Minia, Egypt; d Department of Physics, Faculty of Sciences, Erciyes University, 38039 Kayseri, Turkey; e Department of Chemistry, Faculty of Science, Sohag University, 82524 Sohag, Egypt; f Kirkuk University, College of Science, Department of Chemistry, Kirkuk, Iraq

**Keywords:** crystal structure, indandiones, chromenes, coumarins, hydrogen bonding, π–π stacking

## Abstract

In the title mol­ecule, C_25_H_14_O_4_, the fused-ring system consisting of four rings is approximately planar, with a dihedral angle of 9.62 (5)° between the planes of the indene ring system and the benzene ring. The di­hydro­indene-1,3-dione unit makes a dihedral angle of 63.50 (2)° with the mean plane of the fused-ring system. A weak C—H⋯O inter­action organizes the mol­ecules into a helical chain along the *b* axis. In addition, there is a π–π stacking inter­action between the five-membered rings of adjacent fused-ring systems, with a centroid–centroid distance of 3.666 (1) Å.

## Related literature   

For synthesis and biological properties of chromene scaffolds, see: RamaGanesh *et al.* (2010 ▸); O’Kenedy & Thornes (1997 ▸); Zabradnik (1992 ▸). For the bioactivity of fused chromenes, see: Bargagna *et al.* (1992 ▸); Ermili *et al.* (1979 ▸).
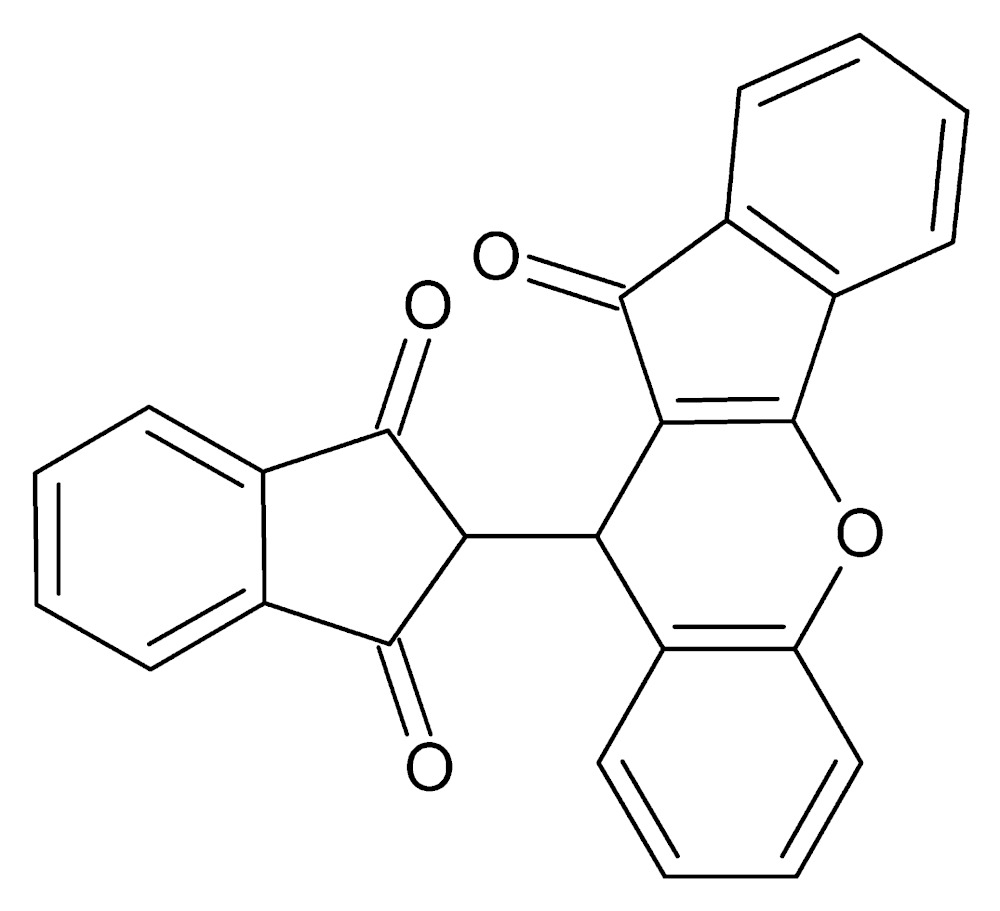


## Experimental   

### Crystal data  

C_25_H_14_O_4_
*M* *_r_* = 378.36
Monoclinic, 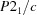

*a* = 8.7409 (2) Å
*b* = 14.4740 (3) Å
*c* = 14.2774 (3) Å
β = 101.141 (1)°
*V* = 1772.28 (7) Å^3^
*Z* = 4
Cu *K*α radiation
μ = 0.78 mm^−1^
*T* = 150 K
0.23 × 0.22 × 0.11 mm

### Data collection  

Bruker D8 VENTURE PHOTON 100 CMOS diffractometer
Absorption correction: multi-scan (*SADABS*; Bruker, 2014 ▸) *T*_min_ = 0.86, *T*_max_ = 0.92
28946 measured reflections
3495 independent reflections
3189 reflections with *I* > 2σ(*I*)
*R*_int_ = 0.030

### Refinement  

*R*[*F* ^2^ > 2σ(*F* ^2^)] = 0.035
*wR*(*F* ^2^) = 0.090
*S* = 1.06
3495 reflections
262 parameters
H-atom parameters constrained
Δρ_max_ = 0.19 e Å^−3^
Δρ_min_ = −0.21 e Å^−3^

### 

Data collection: *APEX2* (Bruker, 2014 ▸); cell refinement: *SAINT* (Bruker, 2014 ▸); data reduction: *SAINT*; program(s) used to solve structure: *SHELXTL* (Sheldrick, 2008 ▸); program(s) used to refine structure: *SHELXL2014* (Sheldrick, 2015 ▸); molecular graphics: *DIAMOND* (Brandenburg & Putz, 2012 ▸); software used to prepare material for publication: *SHELXL2014*.

## Supplementary Material

Crystal structure: contains datablock(s) global, I. DOI: 10.1107/S2056989015007495/is5397sup1.cif

Structure factors: contains datablock(s) I. DOI: 10.1107/S2056989015007495/is5397Isup2.hkl

Supporting information file. DOI: 10.1107/S2056989015007495/is5397Isup3.cml

**Fig. 1.**
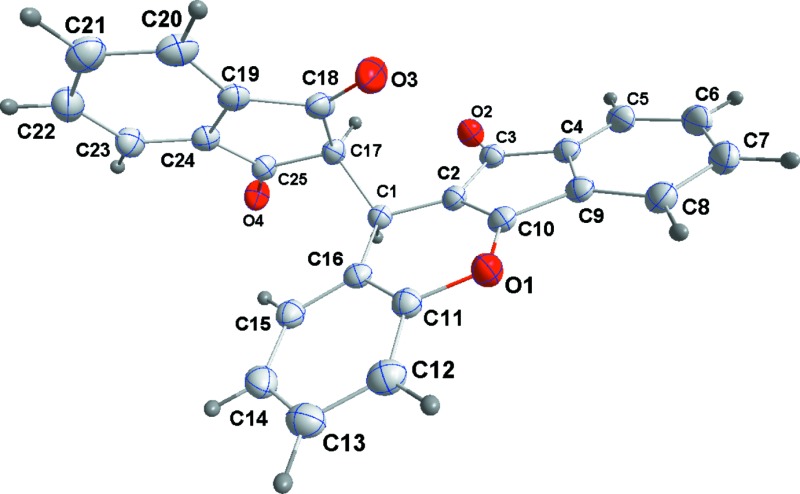
The molecular structure of the title compound with labeling scheme and 50% probability ellipsoids.

. DOI: 10.1107/S2056989015007495/is5397fig1.tif
The mol­ecular structure of the title compound with labeling scheme and 50% probability ellipsoids.

**Fig. 2.**
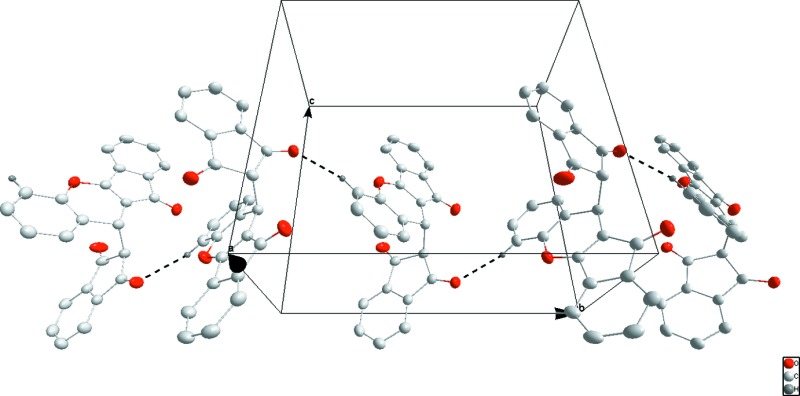
A packing diagram of the title compound, showing a chain structure formed by C—H···O interactions (dashed lines).

. DOI: 10.1107/S2056989015007495/is5397fig2.tif
A packing diagram of the title compound, showing a chain structure formed by C—H⋯O inter­actions (dashed lines).

CCDC reference: 1059989

Additional supporting information:  crystallographic information; 3D view; checkCIF report

## Figures and Tables

**Table 1:** Hydrogen-bond geometry (, )

*D*H*A*	*D*H	H*A*	*D* *A*	*D*H*A*
C12H12O4^i^	0.95	2.54	3.4687(15)	166

Symmetry code: (i) 
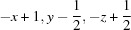
.

## supplementary crystallographic information

### S1. Comment

The synthesis of chromenes scaffolds has attracted considerable attention from organic and medicinal chemists for many years as larg number of natural products contain this heterocyclic nucleus (RamaGanesh *et al.*, 2010). They are widely used as additives in food, perfumes, cosmetics, pharmaceuticals (O'Kenedy & Thornes, 1997), optical brighteners, dispersed fluorescent and laser dyes (Zabradnik, 1992). Fused chromene ring systems have platelet anti-aggregating, local anesthetic (Bargagna *et al.*, 1992) and also exhibit antidepressant effects (Ermili *et al.*, 1979). In this view and following to our study in synthesis of bio-active hetero-cyclic molecules, we report in this study the synthesis and crystal structure of the title compound.

In the title molecule (Fig. 1), there is a slight fold in the larger fused ring moiety along the C1···O1 line with a dihedral angle between the mean planes of C2–C10 and C11–C16 rings being 9.62 (5)°. The dihedral angle between the mean planes of C1–C16/O1 and C17–C25 ring systems is 63.50 (2)°. The molecules associate along the 2_1_ axes *via* a weak C12—H12···O4^i^ [symmetry code: (i) 1-*x*, -1/2+*y*, 1/2-*z*] hydrogen bond to form a helical chain (Table 1 and Fig. 2). In addition, there is a π–π stacking interaction between the five-membered C2–C4/C9/C10 ring and its centrosymmetrically related counterpart with a centroid-centroid distance of 3.666 (1) Å, an interplanar distance of 3.575 (1) Å and a centroid offset of 0.812 (1) Å.

### S2. Experimental

In 30 ml of ethanol, a mixture of 1 mmol (122 mg) of salicylaldehyde and 2 mmol (292 mg) of 1*H*-indene-1,3(2*H*)-dione has been refluxed in the presence of a guanidine derivative as a lewise base catalyst. The reaction was monitored by TLC till completion after 5 h. On cooling, the solid product was collected by filteration, dried under vacuum and recrystallized from dimethylformamide (DMF). Single crystals suitable for X-ray diffraction were obtained by further crystallization from DMF. M.p. 513 K.

### S3. Refinement

H-atoms were placed in calculated positions (C—H = 0.95–1.00 Å) and included as riding contributions with isotropic displacement parameters 1.2 times those of the attached carbon atoms.

### Crystal data

**Table:**

C_25_H_14_O_4_	*F*(000) = 784
*M**_r_* = 378.36	*D*_x_ = 1.418 Mg m^−^^3^
Monoclinic, *P*2_1_/*c*	Cu *K*α radiation, λ = 1.54178 Å
*a* = 8.7409 (2) Å	Cell parameters from 9790 reflections
*b* = 14.4740 (3) Å	θ = 3.1–72.3°
*c* = 14.2774 (3) Å	µ = 0.78 mm^−^^1^
β = 101.141 (1)°	*T* = 150 K
*V* = 1772.28 (7) Å^3^	Block, orange
*Z* = 4	0.23 × 0.22 × 0.11 mm

### Data collection

**Table:**

Bruker D8 VENTURE PHOTON 100 CMOS diffractometer	3495 independent reflections
Radiation source: INCOATEC IµS micro–focus source	3189 reflections with *I* > 2σ(*I*)
Mirror monochromator	*R*_int_ = 0.030
Detector resolution: 10.4167 pixels mm^-1^	θ_max_ = 72.4°, θ_min_ = 4.4°
ω scans	*h* = −10→10
Absorption correction: multi-scan (*SADABS*; Bruker, 2014)	*k* = −17→17
*T*_min_ = 0.86, *T*_max_ = 0.92	*l* = −17→17
28946 measured reflections	

### Refinement

**Table:**

Refinement on *F*^2^	Primary atom site location: structure-invariant direct methods
Least-squares matrix: full	Secondary atom site location: difference Fourier map
*R*[*F*^2^ > 2σ(*F*^2^)] = 0.035	Hydrogen site location: inferred from neighbouring sites
*wR*(*F*^2^) = 0.090	H-atom parameters constrained
*S* = 1.06	*w* = 1/[σ^2^(*F*_o_^2^) + (0.0457*P*)^2^ + 0.5107*P*] where *P* = (*F*_o_^2^ + 2*F*_c_^2^)/3
3495 reflections	(Δ/σ)_max_ < 0.001
262 parameters	Δρ_max_ = 0.19 e Å^−^^3^
0 restraints	Δρ_min_ = −0.21 e Å^−^^3^

### Special details

**Table:**

Geometry. All e.s.d.'s (except the e.s.d. in the dihedral angle between two l.s. planes)are estimated using the full covariance matrix. The cell e.s.d.'s are takeninto account individually in the estimation of e.s.d.'s in distances, anglesand torsion angles; correlations between e.s.d.'s in cell parameters are onlyused when they are defined by crystal symmetry. An approximate (isotropic)treatment of cell e.s.d.'s is used for estimating e.s.d.'s involving l.s. planes.
Refinement. Refinement of *F*^2^ against ALL reflections. The weighted *R*-factor *wR* and goodness of fit *S* are based on *F*^2^, conventional *R*-factors *R* are based on *F*, with *F* set to zero for negative *F*^2^. The threshold expression of *F*^2^ > σ(*F*^2^) is used only for calculating *R*-factors(gt) *etc*. and is not relevant to the choice of reflections for refinement. *R*-factors based on *F*^2^ are statistically about twice as large as those based on *F*, and *R*- factors based on ALL data will be even larger. H-atoms were placed in calculated positions (C—H = 0.95 - 1.00 Å) and included as riding contributions with isotropic displacement parameters 1.2 times those of the attached carbon atoms.

### Fractional atomic coordinates and isotropic or equivalent isotropic displacement parameters (Å^2^)

**Table:**

	*x*	*y*	*z*	*U*_iso_*/*U*_eq_	
O1	0.47506 (10)	0.32965 (6)	0.40020 (6)	0.0302 (2)	
O2	0.16356 (11)	0.59782 (6)	0.38392 (6)	0.0352 (2)	
O3	0.08695 (12)	0.33852 (7)	0.24206 (6)	0.0440 (3)	
O4	0.27958 (11)	0.57260 (6)	0.06450 (6)	0.0343 (2)	
C1	0.35974 (13)	0.48118 (7)	0.26329 (8)	0.0240 (2)	
H1	0.4079	0.5433	0.2586	0.029*	
C2	0.33109 (13)	0.47028 (8)	0.36229 (8)	0.0243 (2)	
C3	0.23649 (13)	0.52859 (8)	0.41347 (8)	0.0263 (2)	
C4	0.24407 (13)	0.48383 (8)	0.50974 (8)	0.0266 (2)	
C5	0.17816 (15)	0.50868 (9)	0.58577 (9)	0.0320 (3)	
H5	0.1152	0.5624	0.5835	0.038*	
C6	0.20691 (15)	0.45208 (10)	0.66709 (9)	0.0354 (3)	
H6	0.1624	0.4677	0.7207	0.043*	
C7	0.29864 (15)	0.37426 (9)	0.67053 (8)	0.0352 (3)	
H7	0.3168	0.3373	0.7266	0.042*	
C8	0.36576 (14)	0.34857 (9)	0.59259 (8)	0.0314 (3)	
H8	0.4289	0.2949	0.5947	0.038*	
C9	0.33631 (13)	0.40432 (8)	0.51294 (8)	0.0260 (2)	
C10	0.38543 (13)	0.39924 (8)	0.42012 (8)	0.0248 (2)	
C11	0.52713 (13)	0.33794 (8)	0.31358 (8)	0.0262 (2)	
C12	0.63660 (14)	0.27212 (8)	0.30077 (9)	0.0314 (3)	
H12	0.6658	0.2247	0.3468	0.038*	
C13	0.70268 (14)	0.27624 (9)	0.22043 (9)	0.0337 (3)	
H13	0.7782	0.2317	0.2110	0.040*	
C14	0.65849 (14)	0.34552 (9)	0.15348 (9)	0.0330 (3)	
H14	0.7039	0.3487	0.0983	0.040*	
C15	0.54789 (14)	0.41008 (8)	0.16735 (8)	0.0291 (3)	
H15	0.5188	0.4572	0.1210	0.035*	
C16	0.47777 (13)	0.40800 (8)	0.24747 (8)	0.0248 (2)	
C17	0.20346 (13)	0.47769 (8)	0.18908 (8)	0.0255 (2)	
H17	0.1298	0.5231	0.2091	0.031*	
C18	0.12395 (13)	0.38332 (8)	0.17816 (8)	0.0280 (3)	
C19	0.10045 (12)	0.35606 (8)	0.07625 (8)	0.0253 (2)	
C20	0.03310 (14)	0.27627 (8)	0.03180 (9)	0.0307 (3)	
H20	−0.0095	0.2302	0.0666	0.037*	
C21	0.03053 (14)	0.26658 (8)	−0.06495 (9)	0.0332 (3)	
H21	−0.0141	0.2126	−0.0969	0.040*	
C22	0.09206 (15)	0.33439 (9)	−0.11675 (9)	0.0334 (3)	
H22	0.0893	0.3255	−0.1830	0.040*	
C23	0.15714 (14)	0.41447 (8)	−0.07265 (8)	0.0303 (3)	
H23	0.1982	0.4610	−0.1077	0.036*	
C24	0.16014 (13)	0.42425 (7)	0.02446 (8)	0.0251 (2)	
C25	0.22245 (13)	0.50203 (8)	0.08798 (8)	0.0258 (2)	

### Atomic displacement parameters (Å^2^)

**Table:**

	*U*^11^	*U*^22^	*U*^33^	*U*^12^	*U*^13^	*U*^23^
O1	0.0330 (4)	0.0288 (4)	0.0296 (4)	0.0069 (3)	0.0081 (3)	0.0060 (3)
O2	0.0399 (5)	0.0292 (4)	0.0385 (5)	0.0078 (4)	0.0125 (4)	0.0058 (4)
O3	0.0468 (6)	0.0541 (6)	0.0294 (5)	−0.0207 (5)	0.0036 (4)	0.0110 (4)
O4	0.0468 (5)	0.0261 (4)	0.0291 (4)	−0.0079 (4)	0.0049 (4)	0.0039 (3)
C1	0.0249 (5)	0.0234 (5)	0.0231 (5)	−0.0017 (4)	0.0033 (4)	0.0008 (4)
C2	0.0236 (5)	0.0248 (5)	0.0234 (5)	−0.0020 (4)	0.0015 (4)	−0.0006 (4)
C3	0.0254 (5)	0.0256 (5)	0.0272 (6)	−0.0028 (4)	0.0037 (4)	−0.0008 (4)
C4	0.0256 (5)	0.0280 (6)	0.0249 (5)	−0.0048 (4)	0.0014 (4)	−0.0016 (4)
C5	0.0331 (6)	0.0334 (6)	0.0296 (6)	−0.0035 (5)	0.0060 (5)	−0.0047 (5)
C6	0.0355 (7)	0.0474 (7)	0.0237 (6)	−0.0090 (6)	0.0065 (5)	−0.0042 (5)
C7	0.0346 (6)	0.0458 (7)	0.0234 (6)	−0.0079 (6)	0.0010 (5)	0.0059 (5)
C8	0.0286 (6)	0.0358 (6)	0.0274 (6)	−0.0023 (5)	−0.0004 (5)	0.0047 (5)
C9	0.0233 (5)	0.0290 (6)	0.0241 (5)	−0.0045 (4)	0.0004 (4)	−0.0006 (4)
C10	0.0221 (5)	0.0255 (5)	0.0255 (5)	−0.0016 (4)	0.0011 (4)	−0.0009 (4)
C11	0.0250 (5)	0.0270 (6)	0.0263 (6)	−0.0018 (4)	0.0039 (4)	−0.0002 (4)
C12	0.0291 (6)	0.0273 (6)	0.0366 (6)	0.0019 (5)	0.0028 (5)	0.0004 (5)
C13	0.0276 (6)	0.0328 (6)	0.0408 (7)	0.0030 (5)	0.0067 (5)	−0.0064 (5)
C14	0.0285 (6)	0.0383 (7)	0.0332 (6)	−0.0027 (5)	0.0088 (5)	−0.0041 (5)
C15	0.0267 (6)	0.0317 (6)	0.0286 (6)	−0.0027 (5)	0.0043 (5)	0.0009 (5)
C16	0.0217 (5)	0.0247 (5)	0.0271 (5)	−0.0033 (4)	0.0022 (4)	−0.0014 (4)
C17	0.0263 (6)	0.0264 (5)	0.0233 (5)	0.0017 (4)	0.0032 (4)	0.0032 (4)
C18	0.0223 (5)	0.0336 (6)	0.0269 (6)	−0.0018 (5)	0.0014 (4)	0.0070 (5)
C19	0.0214 (5)	0.0257 (5)	0.0277 (6)	0.0019 (4)	0.0021 (4)	0.0045 (4)
C20	0.0268 (6)	0.0264 (6)	0.0367 (6)	−0.0022 (5)	0.0010 (5)	0.0059 (5)
C21	0.0324 (6)	0.0259 (6)	0.0387 (7)	−0.0007 (5)	0.0006 (5)	−0.0046 (5)
C22	0.0370 (7)	0.0345 (6)	0.0287 (6)	−0.0001 (5)	0.0063 (5)	−0.0046 (5)
C23	0.0337 (6)	0.0304 (6)	0.0272 (6)	−0.0027 (5)	0.0069 (5)	0.0014 (5)
C24	0.0243 (5)	0.0237 (5)	0.0265 (6)	0.0012 (4)	0.0031 (4)	0.0025 (4)
C25	0.0272 (5)	0.0242 (5)	0.0251 (5)	0.0012 (4)	0.0025 (4)	0.0037 (4)

### Geometric parameters (Å, º)

**Table:**

O1—C10	1.3400 (14)	C11—C16	1.3956 (16)
O1—C11	1.4030 (13)	C12—C13	1.3817 (18)
O2—C3	1.2185 (14)	C12—H12	0.9500
O3—C18	1.2131 (14)	C13—C14	1.3877 (18)
O4—C25	1.2123 (14)	C13—H13	0.9500
C1—C2	1.4906 (15)	C14—C15	1.3861 (17)
C1—C16	1.5256 (15)	C14—H14	0.9500
C1—C17	1.5595 (15)	C15—C16	1.3986 (16)
C1—H1	1.0000	C15—H15	0.9500
C2—C10	1.3464 (16)	C17—C25	1.5256 (15)
C2—C3	1.4709 (16)	C17—C18	1.5268 (16)
C3—C4	1.5093 (16)	C17—H17	1.0000
C4—C5	1.3715 (17)	C18—C19	1.4829 (16)
C4—C9	1.4008 (16)	C19—C20	1.3924 (16)
C5—C6	1.4034 (18)	C19—C24	1.3937 (15)
C5—H5	0.9500	C20—C21	1.3843 (18)
C6—C7	1.378 (2)	C20—H20	0.9500
C6—H6	0.9500	C21—C22	1.3977 (18)
C7—C8	1.4047 (18)	C21—H21	0.9500
C7—H7	0.9500	C22—C23	1.3874 (17)
C8—C9	1.3775 (16)	C22—H22	0.9500
C8—H8	0.9500	C23—C24	1.3888 (16)
C9—C10	1.4717 (15)	C23—H23	0.9500
C11—C12	1.3873 (16)	C24—C25	1.4818 (16)
			
C10—O1—C11	115.17 (9)	C12—C13—H13	120.1
C2—C1—C16	108.01 (9)	C14—C13—H13	120.1
C2—C1—C17	110.81 (9)	C15—C14—C13	119.85 (11)
C16—C1—C17	113.92 (9)	C15—C14—H14	120.1
C2—C1—H1	108.0	C13—C14—H14	120.1
C16—C1—H1	108.0	C14—C15—C16	122.03 (11)
C17—C1—H1	108.0	C14—C15—H15	119.0
C10—C2—C3	107.40 (10)	C16—C15—H15	119.0
C10—C2—C1	123.94 (10)	C11—C16—C15	116.22 (10)
C3—C2—C1	128.60 (10)	C11—C16—C1	122.27 (10)
O2—C3—C2	127.35 (11)	C15—C16—C1	121.46 (10)
O2—C3—C4	126.60 (11)	C25—C17—C18	103.92 (9)
C2—C3—C4	106.03 (9)	C25—C17—C1	113.22 (9)
C5—C4—C9	121.12 (11)	C18—C17—C1	114.84 (9)
C5—C4—C3	131.06 (11)	C25—C17—H17	108.2
C9—C4—C3	107.82 (10)	C18—C17—H17	108.2
C4—C5—C6	117.79 (12)	C1—C17—H17	108.2
C4—C5—H5	121.1	O3—C18—C19	126.10 (11)
C6—C5—H5	121.1	O3—C18—C17	125.71 (11)
C7—C6—C5	121.10 (11)	C19—C18—C17	108.20 (9)
C7—C6—H6	119.4	C20—C19—C24	120.94 (11)
C5—C6—H6	119.4	C20—C19—C18	129.36 (10)
C6—C7—C8	121.16 (11)	C24—C19—C18	109.70 (10)
C6—C7—H7	119.4	C21—C20—C19	117.57 (11)
C8—C7—H7	119.4	C21—C20—H20	121.2
C9—C8—C7	117.31 (12)	C19—C20—H20	121.2
C9—C8—H8	121.3	C20—C21—C22	121.54 (11)
C7—C8—H8	121.3	C20—C21—H21	119.2
C8—C9—C4	121.52 (11)	C22—C21—H21	119.2
C8—C9—C10	132.35 (11)	C23—C22—C21	120.82 (11)
C4—C9—C10	106.13 (10)	C23—C22—H22	119.6
O1—C10—C2	126.54 (10)	C21—C22—H22	119.6
O1—C10—C9	120.86 (10)	C22—C23—C24	117.73 (11)
C2—C10—C9	112.60 (10)	C22—C23—H23	121.1
C12—C11—C16	122.74 (11)	C24—C23—H23	121.1
C12—C11—O1	114.01 (10)	C23—C24—C19	121.38 (11)
C16—C11—O1	123.21 (10)	C23—C24—C25	128.48 (10)
C13—C12—C11	119.30 (11)	C19—C24—C25	110.14 (10)
C13—C12—H12	120.3	O4—C25—C24	126.38 (10)
C11—C12—H12	120.3	O4—C25—C17	125.60 (10)
C12—C13—C14	119.85 (11)	C24—C25—C17	108.02 (9)
			
C16—C1—C2—C10	−8.40 (14)	O1—C11—C16—C15	176.14 (10)
C17—C1—C2—C10	117.03 (12)	C12—C11—C16—C1	−178.73 (10)
C16—C1—C2—C3	174.83 (10)	O1—C11—C16—C1	−1.09 (17)
C17—C1—C2—C3	−59.74 (14)	C14—C15—C16—C11	0.92 (17)
C10—C2—C3—O2	−177.52 (11)	C14—C15—C16—C1	178.17 (10)
C1—C2—C3—O2	−0.33 (19)	C2—C1—C16—C11	7.86 (14)
C10—C2—C3—C4	0.95 (12)	C17—C1—C16—C11	−115.70 (11)
C1—C2—C3—C4	178.15 (10)	C2—C1—C16—C15	−169.22 (10)
O2—C3—C4—C5	−1.7 (2)	C17—C1—C16—C15	67.21 (13)
C2—C3—C4—C5	179.85 (12)	C2—C1—C17—C25	171.83 (9)
O2—C3—C4—C9	178.00 (11)	C16—C1—C17—C25	−66.13 (12)
C2—C3—C4—C9	−0.49 (12)	C2—C1—C17—C18	−69.02 (12)
C9—C4—C5—C6	0.34 (17)	C16—C1—C17—C18	53.01 (13)
C3—C4—C5—C6	179.97 (11)	C25—C17—C18—O3	−179.89 (12)
C4—C5—C6—C7	0.17 (18)	C1—C17—C18—O3	55.90 (16)
C5—C6—C7—C8	−0.39 (19)	C25—C17—C18—C19	0.28 (11)
C6—C7—C8—C9	0.09 (18)	C1—C17—C18—C19	−123.93 (10)
C7—C8—C9—C4	0.42 (17)	O3—C18—C19—C20	0.1 (2)
C7—C8—C9—C10	−179.89 (11)	C17—C18—C19—C20	179.97 (11)
C5—C4—C9—C8	−0.65 (17)	O3—C18—C19—C24	−178.97 (12)
C3—C4—C9—C8	179.64 (10)	C17—C18—C19—C24	0.86 (12)
C5—C4—C9—C10	179.59 (10)	C24—C19—C20—C21	1.22 (17)
C3—C4—C9—C10	−0.12 (12)	C18—C19—C20—C21	−177.80 (11)
C11—O1—C10—C2	6.15 (16)	C19—C20—C21—C22	−0.44 (18)
C11—O1—C10—C9	−173.60 (9)	C20—C21—C22—C23	−0.48 (19)
C3—C2—C10—O1	179.16 (10)	C21—C22—C23—C24	0.59 (18)
C1—C2—C10—O1	1.80 (18)	C22—C23—C24—C19	0.20 (17)
C3—C2—C10—C9	−1.08 (13)	C22—C23—C24—C25	179.92 (11)
C1—C2—C10—C9	−178.44 (10)	C20—C19—C24—C23	−1.13 (17)
C8—C9—C10—O1	0.82 (19)	C18—C19—C24—C23	178.07 (10)
C4—C9—C10—O1	−179.45 (10)	C20—C19—C24—C25	179.10 (10)
C8—C9—C10—C2	−178.95 (12)	C18—C19—C24—C25	−1.70 (13)
C4—C9—C10—C2	0.77 (13)	C23—C24—C25—O4	1.7 (2)
C10—O1—C11—C12	171.53 (10)	C19—C24—C25—O4	−178.55 (11)
C10—O1—C11—C16	−6.30 (15)	C23—C24—C25—C17	−177.87 (11)
C16—C11—C12—C13	1.24 (18)	C19—C24—C25—C17	1.87 (12)
O1—C11—C12—C13	−176.60 (10)	C18—C17—C25—O4	179.17 (11)
C11—C12—C13—C14	−0.34 (18)	C1—C17—C25—O4	−55.58 (15)
C12—C13—C14—C15	−0.22 (18)	C18—C17—C25—C24	−1.26 (11)
C13—C14—C15—C16	−0.09 (18)	C1—C17—C25—C24	124.00 (10)
C12—C11—C16—C15	−1.50 (16)		

### Hydrogen-bond geometry (Å, º)

**Table:**

*D*—H···*A*	*D*—H	H···*A*	*D*···*A*	*D*—H···*A*
C12—H12···O4^i^	0.95	2.54	3.4687 (15)	166

Symmetry code: (i) −*x*+1, *y*−1/2, −*z*+1/2.
