# Supplementary figures and images for: Crystal structure of 2-(11-oxo-10H,11H-indeno­[1,2-b]chromen-10-yl)-2,3-di­hydro-1H-indene-1,3-dione
Source: Acta Crystallogr E Crystallogr Commun. 2015 Apr 22;71(Pt 5):o333–4. doi: 10.1107/S2056989015007495 (PMC4420077; doi:10.1107/S2056989015007495)

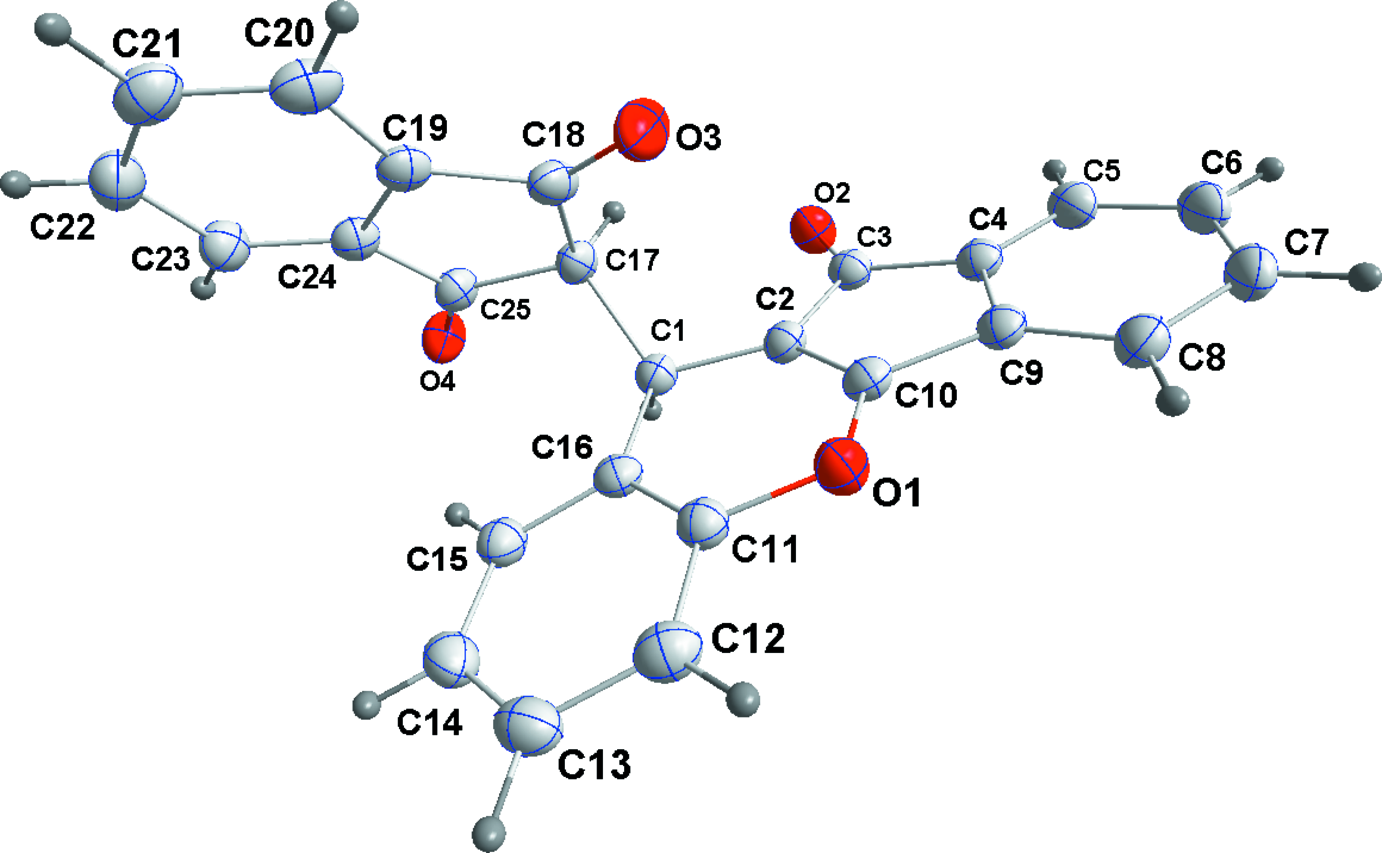

Supplement: Supplementary file 4 [file e-71-0o333-fig1.tif]

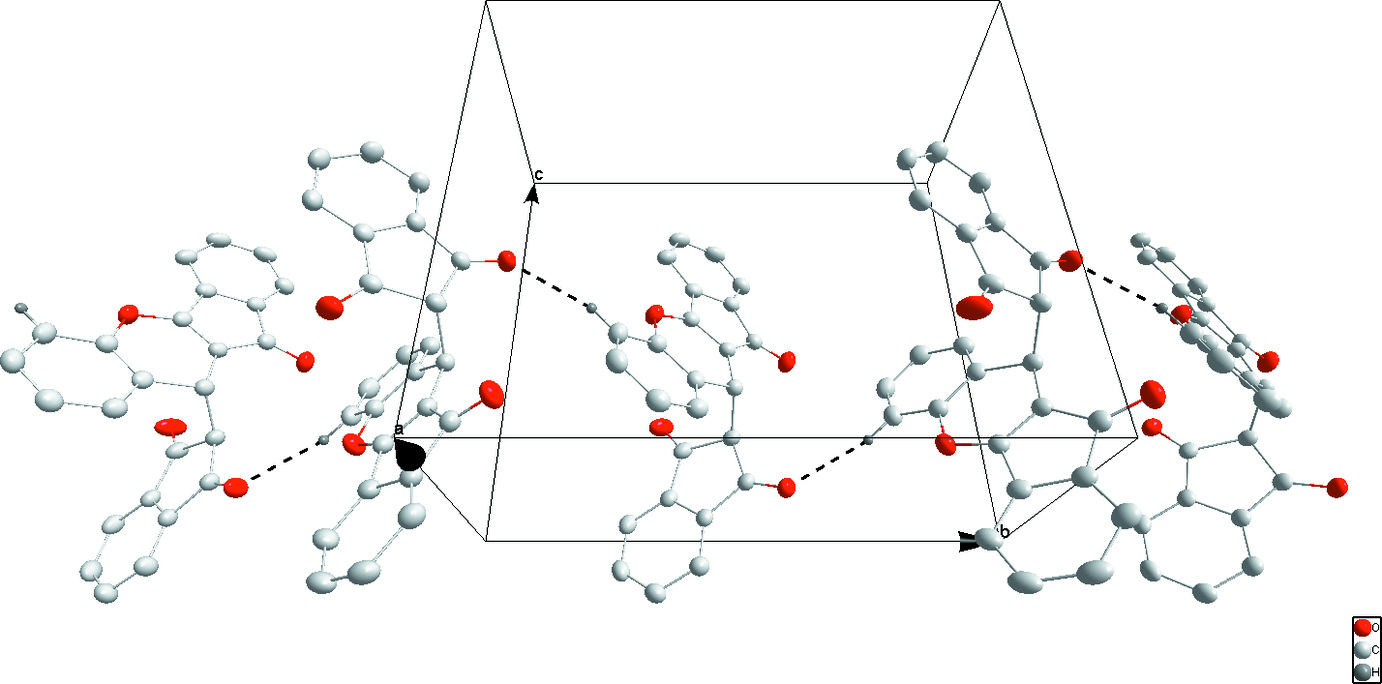

Supplement: Supplementary file 5 [file e-71-0o333-fig2.tif]
